# Genotypic prediction of HIV-1 subtype D tropism

**DOI:** 10.1186/1742-4690-8-56

**Published:** 2011-07-13

**Authors:** Stéphanie Raymond, Pierre Delobel, Marie-Laure Chaix, Michelle Cazabat, Stéphanie Encinas, Patrick Bruel, Karine Sandres-Sauné, Bruno Marchou, Patrice Massip, Jacques Izopet

**Affiliations:** 1 INSERM, U1043, Toulouse, F-31300 France; 2 Université Toulouse III Paul-Sabatier, Faculté de Médecine Toulouse-Purpan, Toulouse, F-31300 France; 3 CHU de Toulouse, Hôpital Purpan, Laboratoire de Virologie, Toulouse, F-31300 France; 4 CHU de Toulouse, Hôpital Purpan, Service des Maladies Infectieuses et Tropicales, Toulouse, F-31300 France; 5 Université Paris Descartes, EA 3620, AP-HP, laboratoire de Virologie, Hôpital Necker-Enfants Malades, Paris, France

## Abstract

**Background:**

HIV-1 subtype D infections have been associated with rapid disease progression and phenotypic assays have shown that CXCR4-using viruses are very prevalent. Recent studies indicate that the genotypic algorithms used routinely to assess HIV-1 tropism may lack accuracy for non-B subtypes. Little is known about the genotypic determinants of HIV-1 subtype D tropism.

**Results:**

We determined the HIV-1 coreceptor usage for 32 patients infected with subtype D by both a recombinant virus phenotypic entry assay and V3-loop sequencing to determine the correlation between them. The sensitivity of the Geno2pheno10 genotypic algorithm was 75% and that of the combined 11/25 and net charge rule was 100% for predicting subtype D CXCR4 usage, but their specificities were poor (54% and 68%). We have identified subtype D determinants in the V3 region associated with CXCR4 use and built a new simple genotypic rule for optimizing the genotypic prediction of HIV-1 subtype D tropism. We validated this algorithm using an independent GenBank data set of 67 subtype D V3 sequences of viruses of known phenotype. The subtype D genotypic algorithm was 68% sensitive and 95% specific for predicting X4 viruses in this data set, approaching the performance of genotypic prediction of HIV-1 subtype B tropism.

**Conclusion:**

The genotypic determinants of HIV-1 subtype D coreceptor usage are slightly different from those for subtype B viruses. Genotypic predictions based on a subtype D-specific algorithm appear to be preferable for characterizing coreceptor usage in epidemiological and pathophysiological studies.

## Background

Human immunodeficiency virus type 1 (HIV-1) enters CD4-expressing cells using one or both of the chemokine receptors CCR5 and CXCR4 [1]. The receptor(s) used by HIV-1 must be identified before a patient is treated with CCR5 antagonists, as these drugs can only be used against R5 viruses alone [2]. Recombinant virus phenotypic entry assays have been widely used to determine HIV-1 tropism [3-5] but genotypic methods based on the V3 sequence could be easier. Several studies indicate that the V3 genotype, combined with bioinformatic algorithms, accurately predicts the phenotype of HIV-1 coreceptor usage for subtype B viruses [6-10]. But the V3-based genotypic algorithms may be unsuitable for predicting the tropism of non-B viruses because they were built using genotype-phenotype correlation data for subtype B viruses [9]. These algorithms can perform differently, as was reported for HIV-1 subtype B [6,10]. The geno2pheno bioinformatic tool accurately predicts subtype C HIV-1 tropism, but is relatively insensitive for predicting CRF02 CXCR4 usage [11,12]. In contrast, the simple rule combining 11/25 and net charge rules accurately predicts HIV-1 tropism for these particular non-B subtypes. The predominant viruses in Uganda and Sudan are subtype D [13]. These subtype D infections are associated with a rapid loss of CD4 cells and disease progression [14-18]. Various phenotypic assays have been used to show that CXCR4-using viruses are very prevalent among subtype D HIV-1 [19-21]. However, little is known about the genotypic determinants of the virus's subtype D tropism [20-23].

This study evaluates the performance of the genotypic algorithms built for subtype B viruses for predicting HIV-1 subtype D tropism. We determined subtype D coreceptor usage with both genotypic and phenotypic assays. The poor concordance between them led us to look for genotypic criteria that could be used to predict the coreceptor usage of subtype D viruses and to define a new genotypic tool for this particular subtype. Lastly, we checked the subtype D genotypic tool against a GenBank data set of subtype D viruses for which both the V3 sequence and the entry phenotype were known.

## Methods

### Study subjects and samples

We studied 32 individuals infected with HIV-1 subtype D recruited at the Department of Infectious Diseases of Toulouse University Hospital, France and at the Department of Virology of Necker-Enfants Malades Hospital, Paris, France. The median age of the patients was 42 years and 46% were men. The median HIV-1 virus load was 4.91 log_10 _copies/ml (IQR [4.1-5.16]). The median CD4 cell count was 355 cells/mm^3 ^(IQR [208.7-634]) and the percentage of CD4 cells was 17% (IQR [10-19.5]). All viruses were identified as HIV-1 subtype D by analysis of the *env *sequence using the NCBI genotyping tool (http://www.ncbi.nlm.nih.gov/projects/genotyping/formpage.cgi). We confirmed that these viruses belonged to the subtype D by neighbor-joining phylogenetic analysis of the sequences studied here, together with HIV-1 subtype reference sequences from the Los Alamos National Laboratory (http://www.hiv.lanl.gov/content/index).

### GenBank data set of HIV-1 subtype D viruses

The V3 sequences of HIV-1 subtype D viruses whose entry phenotype was known were selected from the GenBank database. We selected sequences resulting from bulk sequencing or one sequence per patient in the case of clonal analysis. The entry phenotype of the 67 subtype D viruses had been determined with the MT2 assay or with the Trofile^® ^assay (Monogram, Biosciences).

### Phenotypic characterization of HIV-1 coreceptor usage

We determined the HIV-1 tropism with the TTT phenotypic assay [3]. Briefly, a fragment encompassing the gp120 and the ectodomain of gp41 was amplified by RT-PCR using HIV-1 RNA isolated from the plasma or by PCR from HIV-1 DNA taken from PBMCs. The PCR products then underwent nested PCR. Two amplifications were performed in parallel on aliquots of each sample; the amplified products were then pooled to prevent sampling bias of the virus population.

The phenotype of HIV-1 coreceptor usage was determined using a recombinant virus entry assay with the pNL43-Δenv-Luc2 vector. 293T cells were co-transfected with NheI-linearized pNL43-Δ*env*-Luc2 vector DNA and the product of the nested PCR obtained from the challenged HIV-1-containing sample. The chimeric recombinant virus particles released into the supernatant were used to infect U87 indicator cells bearing CD4 and either CCR5 or CXCR4. Virus entry was assessed by measuring the luciferase activity in lysed cells (as relative light units; RLU). Minor X4 variants were detected when they accounted for 0.5% or more of the total population.

### Genotypic prediction of HIV-1 coreceptor usage

The V3 region was directly sequenced from bulk *env *PCR products in both directions by the dideoxy chain-termination method (BigDye Terminator v3.1; Applied Biosystems, Courtaboeuf, France) on an ABI 3130 DNA sequencer. The two primer pairs used for sequencing have been described [10]. Results were analyzed with Sequencher (Genecodes, Ann Arbor, MI) by an operator blinded to the phenotype. Minority species were detected when the automated sequencer electropherogram showed a second base peak. Multiple alignments were performed with CLUSTALW 1.83, and sequence alignments were manually edited with BioEdit software. Phylogenetic analyses excluded any possibility of sample contamination (data not shown).

We used a combination of criteria from the 11/25 and net charge rules to predict HIV-1 tropism from the V3 genotype [10]. One of the following criteria is required for predicting CXCR4 coreceptor usage: (i) 11 R/K and/or 25 K in V3; (ii) 25 R in V3 and a net charge of ≥ + 5; (iii) a net charge of ≥ +6. The V3 net charge was calculated by subtracting the number of negatively charged amino acids [D and E] from the number of positively charged ones [K and R]. All possible permutations were assessed when amino acid mixtures were found at some codons of V3. The combination resulting in the highest net charge was used to predict the tropism. We also used the geno2pheno tool (with a false positive rate of 10%) to predict HIV-1 coreceptor usage. Geno2pheno is available at http://coreceptor.bioinf.mpi-sb.mpg.de/cgi-bin/coreceptor.pl (September 2010).

### Cloning of *env *PCR products

The *env *PCR products from three patients were subjected to clonal analysis using a TOPO-TA cloning kit (Invitrogen). Plasmids DNA containing *env *inserts were sequenced in the V3 region using the primers previously described [10].

### Statistical methods

The kappa coefficient was measured using STATA SE 9.2 to assess agreement between the genotypic algorithms for HIV-1 tropism prediction and the phenotypic assay. The correlation between two tests is usually considered good when the kappa coefficient is superior to 0.60 with p < 0.05.

### Nucleotide sequence accession numbers

The sequences reported here have been given GenBank accession numbers HQ906854-HQ906879.

## Results

### Phenotypic characterization of HIV-1 subtype D viruses

The *env *products from the plasma sample of 27 of the 32 subtype D-infected patients were successfully amplified by PCR. The phenotype of receptor-mediated entry was then successfully determined for each of these 27 patients. We found 23 virus populations with an R5 phenotype, 2 virus populations with a dual/mixed R5X4 phenotype, and 2 virus populations with a pure X4 phenotype.

### Genotypic prediction of subtype D coreceptor usage with algorithms built for subtype B viruses

**Figure 1:**
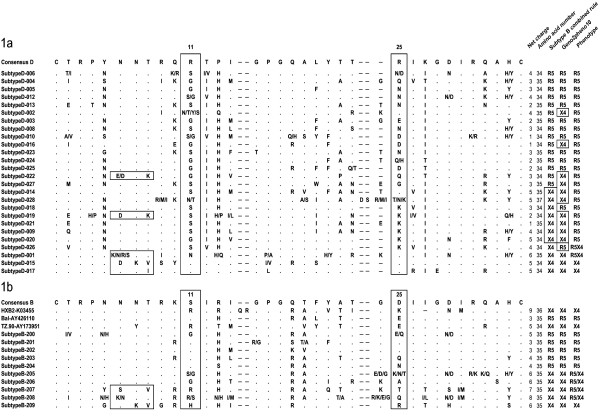
**V3 amino acid sequence alignments and matched phenotypes of the 26 subtype D viruses (1a) and of 13 reference subtype B viruses (1b)**. V3 amino acid sequence alignments were obtained by bulk sequencing *env *PCR products from the 26 subtype D-infected patients, 10 subtype B-infected patients and 3 reference subtype B viruses. These sequences are shown with the following abbreviations with reference to the consensus sequences: dot, identity with amino acid baseline sequence; dash, gap inserted to maintain alignment; slash, amino acid position related to dual virus population. Replacements are indicated by the appropriate code letters. Residues at positions 11 and 25 and mutated N-linked glycosylation sites are boxed to highlight the substitutions noted. The V3 net charge (calculated by subtracting the number of negatively charged amino acids [D and E] from the number of positively charged ones [K and R]); the number of amino acids in V3; the genotype predicted by the combined 11/25 and net charge rules built for subtype B viruses and the Geno2pheno are shown, together with the observed phenotype. Discordances between the genotypic predictions and the phenotype are boxed.

The V3 region was sequenced from the bulk *env *PCR products of the viruses from 26 patients (Figure 1a). We thus obtained genotype-phenotype correlations for 26 patients. The combined 11/25 and net charge rule predicted 15 R5 viruses and 11 X4 viruses, but 7 of them were mis-predicted as X4. Geno2pheno10 predicted 13 R5 viruses and 13 X4 viruses (10 were mis-predicted as X4). As summarized in Table 1 geno2pheno10 was 75% sensitive and 54% specific and the combined rule was 100% sensitive and 68% specific for predicting CXCR4-usage by HIV-1 subtype D. The concordance between the genotypic and phenotypic approaches was 58% with geno2pheno10 and 73% with the combined rule (Table 1).

**Table 1:** Genotypic prediction of HIV-1 subtype D tropism compared to the TTT phenotypic assay

		TTT		Concordance	Genotypic Prediction	
Genotypic tool		R5	R5X4/X4		Sen^a^	Spe^b^
**Geno2pheno10**	**R5**	12	1	58%	75%	54%
	**X4**	10	3	κ = 0.15 (p = 0.14)		
**Combined 11/25 and net charge rules**	**R5**	15	0	73%	100%	68%
	**X4**	7	4	κ = 0.40 (p < 0.01)		

^a^Sen: sensitivity is the capacity for detecting CXCR4-using viruses, calculated by the number of concordant X4/R5X4 results divided by the number of viruses phenotyped as R5X4/X4.

^b^Spe: specificity is the capacity for detecting exclusive CCR5-using viruses, calculated by the number of concordant R5 results divided by the number of viruses phenotyped as R5.

κ: kappa coefficient.

### Genotypic determinants predicting CXCR4 use by HIV-1 subtype D viruses

**Figure 2:**
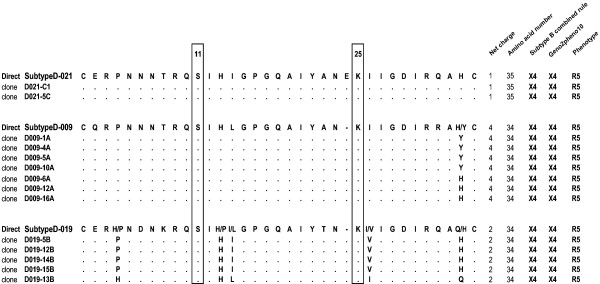
**Clonal analysis of the virus populations of three patients whose genotypic prediction and phenotype were discordant**. Clonal composition of the HIV-1 quasispecies of three patients harboring R5 phenotyped viruses mispredicted X4 by the genotypic algorithms built for subtype B viruses. V3 amino acid sequence alignments were obtained by sequencing molecular clones of *env *PCR products. Theses sequences are shown with the following abbreviations with reference to the direct sequence: dot, identity with amino acid baseline sequence; dash, gap inserted to maintain alignment; slash, amino acid position related to dual virus population. Replacements are indicated by the appropriate code letters. Residues at positions 11 and 25 are boxed to highlight the substitutions noted. The V3 net charge (calculated by subtracting the number of negatively charged amino acids [D and E] from the number of positively charged ones [K and R]); the number of amino acids in V3; the genotype predicted by the combined 11/25 and net charge rules built for subtype B viruses and the Geno2pheno are shown, together with the observed phenotype.

We looked for V3 genotypic determinants known to be associated with CXCR4 usage by subtype B viruses, as shown in Figure 1b[24,25]. One virus had an arginine (R) at position 25 with a net charge at +6 and had an R5X4 phenotype (Figure 1a and Table 2). Eleven viruses had no "R" or "K" at positions 11 or 25, net charges < +5, and R5 phenotypes. Five viruses each had a lysine (K) at position 25 with a net charge < +5, four of which had an R5 phenotype and only one of which had an R5X4 phenotype. Two viruses each had a K at position 25 with a net charge of +5 and were phenotyped as R5. We studied different clones from the HIV-1 quasispecies of three patients harboring R5 virus populations on bulk phenotypic analysis but predicted to be X4 by the bulk genotypic analysis when using the algorithms built for subtype B viruses. All the clones successfully phenotyped were R5 and had a lysine (K) at position 25 with net charges comprised between +1 and +4 (Figure 2). Thus, HIV-1 subtype D viruses frequently have a lysine at position 25 and viruses use exclusively CCR5 for entry when this amino acid is associated with a V3 net charge ≤ +5.

**Table 2:** Genotypic determinants predicting coreceptor use by HIV-1 subtype D viruses

Criteria observed in V3		No. of Bulk Sequences with the Indicated Phenotype (TTT)	
		
11/25 Amino Acids	Net charge	R5	R5X4/X4
"R" or "K" at 11	< +5	0	0
	≥ +5	0	2*
			
"K" at 25	< +5	4	1
	= +5	2	0
	> +5	0	0
			
"R" at 25	< +5	1	0
	≥ +5	1	3*
			
No "R" or "K" at 11 or 25	< +6	14	0
	≥ +6	0	0

*The two viruses harboring an arginine at position 11 have also an arginine at position 25.

We, therefore, designed a genotypic rule based on the 11/25 and net charge rules for determining the tropism of HIV-1 subtype D. One of the following criteria was required for predicting subtype D CXCR4 coreceptor usage: (i) R or K at position 11 of V3; (ii) R at position 25 of V3 and a net charge of ≥ +5; (iii) a net charge of ≥ +6. The genotypic and phenotypic approaches using this rule were 92% concordant (Table 3). This subtype D genotypic algorithm was 75% sensitive and 95% specific with our data set.

**Table 3:** Genotypic prediction of HIV-1 tropism by a subtype D specific algorithm compared to the observed phenotype

		TTT		Concordance	Genotypic Prediction	
Genotypic tool		R5	R5X4/X4		Sen^a^	Spe^b^
**Combined 11/25 and net charge rules for subtype D**	**R5**	21	1	92%	75%	95%
	**X4**	1	3	κ = 0.70 (p < 0.001)		

^a^Sen: sensitivity for predicting CXCR4-using viruses

^b^Spe: specificity for predicting CXCR4-using viruses

κ: kappa coefficient.

### Validation of the subtype D genotypic algorithm on an independent data set

**Figure 3:**
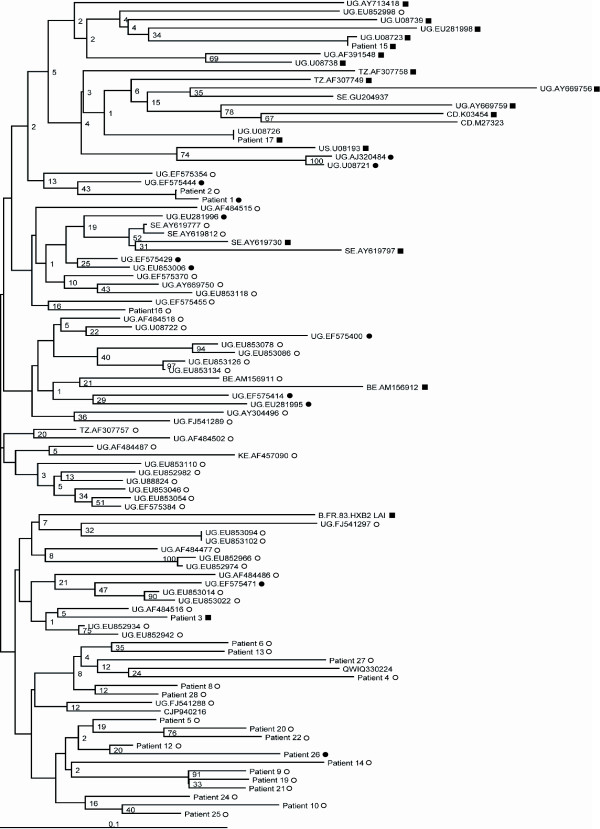
**Neighbour-joining phylogenetic tree of HIV-1 subtype D V3 sequences from 26 patients and 72 sequences from GenBank**. Patients are identified with the same number than in Figure 1 and the GenBank sequences are identified with the country (two letters code) and the accession number. The corresponding phenotype is indicated by symbols: open circles indicate sequences from R5 viruses, solid circles indicate sequences from R5X4 viruses and solid squares indicate sequences from X4 viruses. Percentage bootstrap values are indicated on branches have been calculated for 1000 replicates. The genetic relatedness of two different sequences is represented by the horizontal distance that separates them, with the length of the bar at the bottom denoting a sequence divergence of 0.10.

The GenBank dataset of subtype D viruses included 25 R5X4/X4 viruses and 42 R5 viruses based on phenotypic assays. We analyzed phylogenetically the GenBank V3 sequences and the 26 V3 sequences from patients (Figure 3). We predicted the tropism of these viruses with the initially validated combined rule [10], with the geno2pheno tool and with the subtype D genotypic algorithm based on the simple 11/25 and net charge rules (Table 4). The concordance between genotypic and phenotypic determinations was 69% with the combined rule and 67% with geno2pheno10. The concordance increased to 85% using the subtype D genotypic algorithm. The subtype D tool was 68% sensitive and 95% specific for detecting CXCR4-using viruses. Geno2pheno10 was the most sensitive tool (96%) but its specificity was poor (50%).

**Table 4:** Comparison of genotypic prediction of HIV tropism and the observed phenotype on a GenBank data set of HIV-1 subtype D viruses

		Phenotype		Concordance	Genotypic Prediction	
Genotypic tool		R5	R5X4/X4		Sen^a^	Spe^b^
**Geno2pheno10**	**R5**	21	1	67%	96%	50%
	**X4**	21	24	κ = 0.40 (p < 0.001)		
						
**Combined 11/25 and net charge rules**	**R5**	26	5	69%	80%	62%
	**X4**	16	20	κ = 0.38 (p < 0.001)		
						
**Combined 11/25 and net charge rules for subtype D**	**R5**	40	8	85%	68%	95%
	**X4**	2	17	κ = 0.63 (p < 0.0001)		

^a^Sen: sensitivity for predicting CXCR4-using viruses

^b^Spe: specificity for predicting CXCR4-using viruses

κ: kappa coefficient.

## Discussion

HIV-1 subtype D infections have been associated with rapid disease progression [14-16,18] and a high prevalence of CXCR4-using viruses according to phenotypic assays [19-21]. A genotypic assay for determining subtype D tropism would be useful for investigating the pathogenesis of this subtype and for facilitating the clinical use of CCR5 antagonists. But recent studies indicate that the genotypic algorithms currently used are relatively insensitive for non-B subtypes, although their performance for particular subtypes was not specifically determined [9]. We have now analyzed the correlations between phenotypic and genotypic approaches for determining HIV-1 subtype D tropism.

The prevalence of X4 viruses estimated in 26 patients with the TTT phenotypic assay was 15%. The TTT assay has previously been validated on B and non-B subtypes and correlated well with the enhanced sensitive Trofile assay [3,26]. The scarcity of CXCR4-using viruses in these patients could be because they were at a different stage of the disease compared to the patients recruited in Uganda and Sudan in previous studies [19-21]. The genotypic determination of HIV-1 tropism with algorithms built for the subtype B were adequately sensitive (75% with geno2pheno10 and 100% with the combined 11/25 and net charge rules), but were poorly specific (54% to 68%, respectively) for predicting CXCR4 use by subtype D viruses. One previous study of the performance of two genotypic algorithms for determining subtype D tropism reported poor specificity, 74% for the 11 RK/25 K rule and 53% for the PSSM algorithm [20].

We therefore analyzed the V3-loop sequences and the corresponding phenotype of these subtype D viruses. We found that the lysine at position 25 is a polymorphic amino acid in HIV-1 subtype D and should not be considered as a determinant of CXCR4 usage for this particular subtype, in the contrary with subtype B viruses. We confirmed this polymorphism at a clonal level on three virus populations phenotyped R5. The V1-V2 env region may also influence the virus tropism [27-29]. A recent study found that analysis of the V2-V3 region slightly improved the sensitivity for predicting CXCR4 usage compared to analysis of V3 alone [30]. However, we previously analyzed the V1 and V2 regions of subtype B viruses and found no criteria that improved the genotypic prediction [8]. For subtype D viruses, no genotypic determinant has been identified in the V1-V2 region that improves the performance of the genotypic approaches (data not shown). The determinants identified for predicting CXCR4 usage by subtype D viruses were combined in a simple genotypic rule that differed slightly from the combined rule validated for subtype B, C and CRF02-AG [10-12]. The concordance between the subtype D genotypic rule and the TTT (kappa coefficient: 0.70) was better than that for the subtype B tools (kappa coefficients: 0.15-0.40). The sensitivity of the subtype D genotypic rule (75%) was similar to that of the subtype B genotypic algorithms, applied to subtype B viruses to determine tropism (69 to 88%) [10].

One limit of the study was the small number of X4 viruses in our patients (4/26). However, R5 viruses with an X4 genotype using current algorithms were very informative and analysis of our dataset enabled us to propose a new interpretation rule for HIV-1 subtype D tropism. This new rule was subsequently validated by examination of a GenBank set of 67 subtype D V3 sequences belonging to viruses whose phenotype was known. The best concordance with the phenotype was obtained with the subtype D combined rule (sensitivity 68%, specificity 95%), giving a good agreement with the phenotypic assay (kappa coefficient of 0.63). This specific genotypic algorithm predicted HIV-1 tropism better than did the MT2 or Trofile phenotypic assays (data not shown). The specificity of the V3 genotype is important for not excluding patients eligible for antiretroviral treatment based on a CCR5 antagonist and for epidemiological and pathophysiological studies. The specificity of the V3 genotype is also crucial for accurate characterization of HIV-1 quasispecies by ultra-deep pyrosequencing, which improves the sensitivity for detecting CXCR4-using viruses.

## Conclusion

The combined rule with criteria from the 11/25 and net charge rules modified for subtype D HIV-1 performed well for predicting the tropism of this particular subtype. Simple genotypic methods could make it easier to determine the impact of virus tropism on disease progression and to facilitate the clinical use of CCR5 antagonists. Further studies are now needed to optimize the various genotypic algorithms for predicting the tropism of other HIV-1 non-B subtypes.

## Competing interests

The authors declare that they have no competing interests.

## Authors' contributions

SR, PD and JI assisted with manuscript writing; MLC, BM and PM assisted with patients' care and data acquisition; MC, SE and PB assisted with laboratory assays; KSS assisted with methodological approach; JI and PD assisted with research group leading. All authors read and approved the final manuscript.

## References

[B1] Berger EA Murphy PM Farber JM Chemokine receptors as HIV-1 coreceptors: roles in viral entry, tropism, and disease Annu Rev Immunol 1999 17 657 700 10.1146/annurev.immunol.17.1.657 10358771

[B2] Dorr P Westby M Dobbs S Griffin P Irvine B Macartney M Mori J Rickett G Smith-Burchnell C Napier C Maraviroc (UK-427,857), a potent, orally bioavailable, and selective small-molecule inhibitor of chemokine receptor CCR5 with broad-spectrum anti-human immunodeficiency virus type 1 activity Antimicrob Agents Chemother 2005 49 4721 4732 10.1128/AAC.49.11.4721-4732.2005 16251317 PMC1280117

[B3] Raymond S Delobel P Mavigner M Cazabat M Souyris C Encinas S Bruel P Sandres-Saune K Marchou B Massip P Izopet J Development and performance of a new recombinant virus phenotypic entry assay to determine HIV-1 coreceptor usage J Clin Virol 2010 47 126 130 10.1016/j.jcv.2009.11.018 20015684

[B4] Trouplin V Salvatori F Cappello F Obry V Brelot A Heveker N Alizon M Scarlatti G Clavel F Mammano F Determination of coreceptor usage of human immunodeficiency virus type 1 from patient plasma samples by using a recombinant phenotypic assay J Virol 2001 75 251 259 10.1128/JVI.75.1.251-259.2001 11119595 PMC113919

[B5] Whitcomb JM Huang W Fransen S Limoli K Toma J Wrin T Chappey C Kiss LD Paxinos EE Petropoulos CJ Development and characterization of a novel single-cycle recombinant-virus assay to determine human immunodeficiency virus type 1 coreceptor tropism Antimicrob Agents Chemother 2007 51 566 575 10.1128/AAC.00853-06 17116663 PMC1797738

[B6] Recordon-Pinson P Soulie C Flandre P Descamps D Lazrek M Charpentier C Montes B Trabaud MA Cottalorda J Schneider V Evaluation of the genotypic prediction of HIV-1 coreceptor use versus a phenotypic assay and correlation with the virological response to maraviroc: the ANRS GenoTropism study Antimicrob Agents Chemother 2010 54 3335 3340 10.1128/AAC.00148-10 20530226 PMC2916345

[B7] Seclen E Garrido C Gonzalez Mdel M Gonzalez-Lahoz J de Mendoza C Soriano V Poveda E High sensitivity of specific genotypic tools for detection of X4 variants in antiretroviral-experienced patients suitable to be treated with CCR5 antagonists J Antimicrob Chemother 2010 65 1486 1492 10.1093/jac/dkq137 20427374

[B8] Delobel P Nugeyre MT Cazabat M Pasquier C Marchou B Massip P Barre-Sinoussi F Israel N Izopet J Population-based sequencing of the V3 region of env for predicting the coreceptor usage of human immunodeficiency virus type 1 quasispecies J Clin Microbiol 2007 45 1572 1580 10.1128/JCM.02090-06 17329448 PMC1865905

[B9] Garrido C Roulet V Chueca N Poveda E Aguilera A Skrabal K Zahonero N Carlos S Garcia F Faudon JL Evaluation of eight different bioinformatics tools to predict viral tropism in different human immunodeficiency virus type 1 subtypes J Clin Microbiol 2008 46 887 891 10.1128/JCM.01611-07 18199789 PMC2268339

[B10] Raymond S Delobel P Mavigner M Cazabat M Souyris C Sandres-Saune K Cuzin L Marchou B Massip P Izopet J Correlation between genotypic predictions based on V3 sequences and phenotypic determination of HIV-1 tropism Aids 2008 22 F11 16 10.1097/QAD.0b013e32830ebcd4 18753930

[B11] Raymond S Delobel P Mavigner M Cazabat M Souyris C Encinas S Sandres-Saune K Pasquier C Marchou B Massip P Izopet J Genotypic prediction of human immunodeficiency virus type 1 CRF02-AG tropism J Clin Microbiol 2009 47 2292 2294 10.1128/JCM.02439-08 19439544 PMC2708518

[B12] Raymond S Delobel P Mavigner M Ferradini L Cazabat M Souyris C Sandres-Saune K Pasquier C Marchou B Massip P Izopet J Prediction of HIV type 1 subtype C tropism by genotypic algorithms built from subtype B viruses J Acquir Immune Defic Syndr 2010 53 167 175 10.1097/QAI.0b013e3181c8413b 19996764

[B13] Taylor BS Hammer SM The challenge of HIV-1 subtype diversity N Engl J Med 2008 359 1965 1966 10.1056/NEJMc086373 18971501

[B14] Baeten JM Chohan B Lavreys L Chohan V McClelland RS Certain L Mandaliya K Jaoko W Overbaugh J HIV-1 subtype D infection is associated with faster disease progression than subtype A in spite of similar plasma HIV-1 loads J Infect Dis 2007 195 1177 1180 10.1086/512682 17357054

[B15] Easterbrook PJ Smith M Mullen J O'Shea S Chrystie I de Ruiter A Tatt ID Geretti AM Zuckerman M Impact of HIV-1 viral subtype on disease progression and response to antiretroviral therapy J Int AIDS Soc 2010 13 4 10.1186/1758-2652-13-4 20205896 PMC2827379

[B16] Kaleebu P French N Mahe C Yirrell D Watera C Lyagoba F Nakiyingi J Rutebemberwa A Morgan D Weber J Effect of human immunodeficiency virus (HIV) type 1 envelope subtypes A and D on disease progression in a large cohort of HIV-1-positive persons in Uganda J Infect Dis 2002 185 1244 1250 10.1086/340130 12001041

[B17] Kiwanuka N Robb M Laeyendecker O Kigozi G Wabwire-Mangen F Makumbi FE Nalugoda F Kagaayi J Eller M Eller LA HIV-1 viral subtype differences in the rate of CD4+ T-cell decline among HIV seroincident antiretroviral naive persons in Rakai district, Uganda J Acquir Immune Defic Syndr 2010 54 180 184 20010433 10.1097/QAI.0b013e3181c98fc0 PMC2877752

[B18] Vasan A Renjifo B Hertzmark E Chaplin B Msamanga G Essex M Fawzi W Hunter D Different rates of disease progression of HIV type 1 infection in Tanzania based on infecting subtype Clin Infect Dis 2006 42 843 852 10.1086/499952 16477563

[B19] Church JD Huang W Mwatha A Toma J Stawiski E Donnell D Guay LA Mmiro F Musoke P Jackson JB HIV-1 tropism and survival in vertically infected Ugandan infants J Infect Dis 2008 197 1382 1388 10.1086/587492 18444795

[B20] Huang W Eshleman SH Toma J Fransen S Stawiski E Paxinos EE Whitcomb JM Young AM Donnell D Mmiro F Coreceptor tropism in human immunodeficiency virus type 1 subtype D: high prevalence of CXCR4 tropism and heterogeneous composition of viral populations J Virol 2007 81 7885 7893 10.1128/JVI.00218-07 17507467 PMC1951291

[B21] Kaleebu P Nankya IL Yirrell DL Shafer LA Kyosiimire-Lugemwa J Lule DB Morgan D Beddows S Weber J Whitworth JA Relation between chemokine receptor use, disease stage, and HIV-1 subtypes A and D: results from a rural Ugandan cohort J Acquir Immune Defic Syndr 2007 45 28 33 10.1097/QAI.0b013e3180385aa0 17310935

[B22] De Wolf F Hogervorst E Goudsmit J Fenyo EM Rubsamen-Waigmann H Holmes H Galvao-Castro B Karita E Wasi C Sempala SD Syncytium-inducing and non-syncytium-inducing capacity of human immunodeficiency virus type 1 subtypes other than B: phenotypic and genotypic characteristics. WHO Network for HIV Isolation and Characterization AIDS Res Hum Retroviruses 1994 10 1387 1400 10.1089/aid.1994.10.1387 7888192

[B23] Zhong P Peeters M Janssens W Fransen K Heyndrickx L Vanham G Willems B Piot P van der Groen G Correlation between genetic and biological properties of biologically cloned HIV type 1 viruses representing subtypes A, B, and D AIDS Res Hum Retroviruses 1995 11 239 248 10.1089/aid.1995.11.239 7742038

[B24] De Jong JJ De Ronde A Keulen W Tersmette M Goudsmit J Minimal requirements for the human immunodeficiency virus type 1 V3 domain to support the syncytium-inducing phenotype: analysis by single amino acid substitution J Virol 1992 66 6777 6780 1404617 10.1128/jvi.66.11.6777-6780.1992 PMC240176

[B25] Fouchier RA Groenink M Kootstra NA Tersmette M Huisman HG Miedema F Schuitemaker H Phenotype-associated sequence variation in the third variable domain of the human immunodeficiency virus type 1 gp120 molecule J Virol 1992 66 3183 3187 1560543 10.1128/jvi.66.5.3183-3187.1992 PMC241084

[B26] Saliou A Delobel P Dubois M Nicot F Raymond S Calvez V Masquelier B Izopet J Concordance between Two Phenotypic Assays and Ultradeep Pyrosequencing for Determining HIV-1 Tropism Antimicrob Agents Chemother 2011 55 2831 2836 10.1128/AAC.00091-11 21464245 PMC3101380

[B27] Carrillo A Ratner L Cooperative effects of the human immunodeficiency virus type 1 envelope variable loops V1 and V3 in mediating infectivity for T cells J Virol 1996 70 1310 1316 8551601 10.1128/jvi.70.2.1310-1316.1996 PMC189949

[B28] Masciotra S Owen SM Rudolph D Yang C Wang B Saksena N Spira T Dhawan S Lal RB Temporal relationship between V1V2 variation, macrophage replication, and coreceptor adaptation during HIV-1 disease progression AIDS 2002 16 1887 1898 10.1097/00002030-200209270-00005 12351948

[B29] Groenink M Andeweg AC Fouchier RA Broersen S van der Jagt RC Schuitemaker H de Goede RE Bosch ML Huisman HG Tersmette M Phenotype-associated env gene variation among eight related human immunodeficiency virus type 1 clones: evidence for in vivo recombination and determinants of cytotropism outside the V3 domain J Virol 1992 66 6175 6180 1527855 10.1128/jvi.66.10.6175-6180.1992 PMC283667

[B30] Thielen A Sichtig N Kaiser R Lam J Harrigan PR Lengauer T Improved prediction of HIV-1 coreceptor usage with sequence information from the second hypervariable loop of gp120 J Infect Dis 2010 202 1435 1443 10.1086/656600 20874088

